# Resolution of fluoroquinolone-resistant *Escherichia coli* keratitis with a PROSE device for enhanced targeted antibiotic delivery

**DOI:** 10.1016/j.ajoc.2018.09.006

**Published:** 2018-09-12

**Authors:** Hualei Zhai, Paulo J.M. Bispo, Hidenaga Kobashi, Deborah S. Jacobs, Michael S. Gilmore, Joseph B. Ciolino

**Affiliations:** a Department of Ophthalmology, Massachusetts Eye and Ear, Harvard Medical School, Boston, USA; b Infectious Diseases Institute, Massachusetts Eye and Ear, Harvard Medical School, Boston, USA; c BostonSight, Needham, MA, USA

**Keywords:** *Escherichia coli*, Bacterial keratitis, Fluoroquinolone, Antibiotic resistance, Prosthetic replacement of the ocular surface ecosystem (PROSE)

## Abstract

**Purpose:**

To report the resolution of a fluoroquinolone-resistant *Escherichia coli* keratitis with use of a prosthetic replacement of the ocular surface ecosystem (PROSE) device for enhanced targeted delivery of moxifloxiacin.

**Observations:**

A 62-year-old female presented with a 3-day history of pain, photophobia, and declining vision in left eye. The patient had a 2-year history of binocular PROSE treatment for ocular chronic graft-vs-host disease (cGVHD). A corneal ulcer was diagnosed and treated with topical 0.5% moxifloxacin solution 6 times per day, with continued wear of the PROSE device. After 4 days, worsening symptoms led to an increase in application of moxifloxicin to every 2 hours while awake. The drug was administered by removal of the device, cleaning and replenishing the reservoir with sterile saline, and adding one drop of the drug to the reservoir prior to reinsertion. Four days later, the corneal surface was epithelialized with only small subepithelial infiltrate remaining. The corneal culture grew an *E. coli* isolate carrying multiple mutations in the topoisomerase genes. These mutations were correlated with varying levels of resistance to ciprofloxacin (256 μg/mL), levofloxacin (8 μg/mL), and moxifloxacin (16 μg/mL).

**Conclusions and Importance:**

Although the infecting *E. coli* strain exhibited resistance to fluoroquinolones, the infection resolved when moxifloxacin was combined with PROSE therapy. Frequent dosing to the PROSE reservoir is likely to increase fluoroquinolone bioavailability and may represent a valuable approach to overcome antibiotic resistance.

## 1 Introduction

Antibiotic resistance is a global health problem, and antibiotic resistant bacteria are emerging faster than the development of new antibiotics.^1^^,^^2^ To limit the emergence of resistance, new therapies that target effective doses of antimicrobials to sites of infection, and limit collateral exposure of the human microbiome are needed. Some cases of antibiotic resistance can be overcome with sustained antibiotic delivery to maximize concentrations in the infected tissue that expose the organisms to levels of antibiotics that may exceed the minimal inhibitory concentration (MIC) value and exert bactericidal effect.^3^

**Fig. 1:**
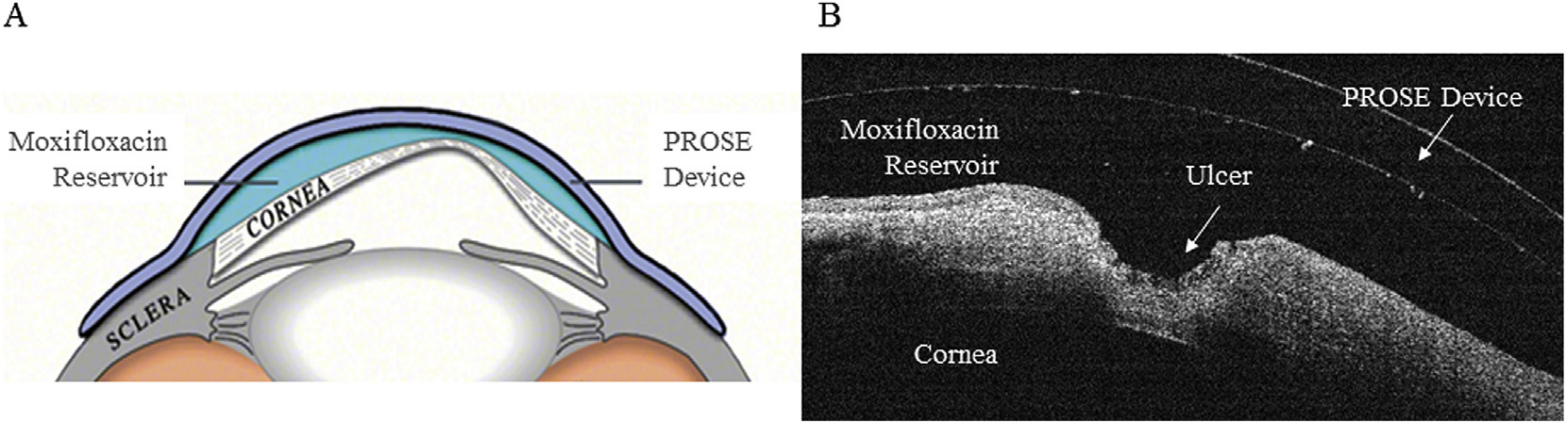
Reservoir compartment created by the PROSE lens. A) Schematic design of PROSE device and B) ocular coherence tomography image of a reservoir of tears containing moxifloxacin between PROSE and cornea that has an ulcer with focal thinning.

The scleral lenses used in prosthetic replacement of the ocular surface ecosystem (PROSE, BostonSight, Needham MA) treatment offer a mechanism for providing extended, targeted exposure of drugs to the ocular surface. The PROSE device creates a reservoir of artificial tears at the ocular surface (Fig. 1A), supports healing, reduces symptoms, restores vision, and improves quality of life for patients with complex corneal disease. Prior studies have shown the safety and efficacy of PROSE treatment and other scleral lenses in patients with graft-vs-host disease (GVHD).^4^, ^5^, ^6^ In addition, there have been reports of treatment with the PROSE device to increase the bioavailability of a VEGF inhibitor for the treatment of active corneal neovascularization.^7^

Here we report a patient with a history of chronic GVHD (cGVDH), who developed a sight-threatening central corneal ulcer caused by a fluoroquinolone-resistant *E. coli* strain, which resolved using the PROSE device for enhanced targeted delivery of moxifloxacin to the ocular surface.

## 2 Case report

A 62-year-old woman was referred to the authors' hospital for evaluation of a corneal ulcer in her left eye. With the patient's consent, a review of the clinic record was conducted. Ten years previously she received an allogeneic hematopoetic stem cell transplant for treatment of myelodysplasia syndrome. Her course was complicated by the development of cGVHD affecting the liver, skin, esophagus, mouth, and eyes. Severe keratoconjunctivitis sicca had been treated with a PROSE lens for more than 2 years prior to developing this corneal ulcer. Her medical status was compromised by systemic steroid dependence and by steroid induced diabetes mellitus.

After presenting with a corneal ulcer in her left eye, the patient was empirically treated with topical 0.5% moxifloxacin (Vigamox, Alcon) that was applied 6 times a day (once before the PROSE lens was inserted in the morning, 4 times during the day inside of the PROSE lens reservoir, and once again at night after the PROSE lens was removed). After failure to improve on 4 days of this treatment, she was referred to Massachusetts Eye and Ear Infirmary for additional corneal ulcer evaluation, culture and modification of treatment. Upon presentation, the central cornea was opaque and neovascularized with tissue loss of approximately 60% of the corneal thickness. There was an epithelial defect that measured 2.5 mm by 1 mm, with an underlying 2 mm by 1 mm infiltrate. Microbiological smears and cultures were performed with the specimen from corneal scraping. No bacteria or fungi were evident with Gram stain or Calcofluor white stain. At the time, frequency of application of topical moxifloxacin was increased to every 2 hours while awake (approximately 8 times per day), delivered as one drop added to the PROSE reservoir after removal and cleaning reinsertion of the device and replenishment of the reservoir with preservative free saline (Fig. 1B).

Four days after culture and modification of antibiotic delivery regimen, the corneal ulcer resolved, with re-epithelialization of the cornea surface and resolution of the infiltrate. A strain of *E. coli* cultured from the lesion in 5% sheep blood agar media exhibited resistance to fluoroquinolones, trimethoprim/sulfamethoxazole and ampicillin and ampicillin-sulbactam according to breakpoints set by the Clinical Laboratory Standard Institute-CLSI^8^ (Table 1). The strain carried multiple single point mutations in the quinolone resistance-determining region (QRDR) of *gyr*A (Ser83Leu, Asp87Asn), *par*C (Ser57Thr, Ser80Ile) and *par*E (Leu416Phe) genes. These mutations were correlated with varying levels of resistance to the fluoroquinolones: ciprofloxacin (MIC 256 μg/mL), levofloxacin (8 μg/mL) and moxifloxacin (16 μg/mL), as determined by reference broth microdilution.

**Table 1:** Antimicrobial susceptibility profile of the fluoroquinolone-resistant *E. coli* isolate as determined by the MicroScanWalk Away system.

Antibiotic	Sensitivity
Ciprofloxacin	Resistant
Levofloxacin	Resistant
Moxifloxacin	Resistant^a^
Amikacin	Susceptible
Ampicillin	Resistant
Ampicillin-sulbactam	Resistant
Aztreonam	Susceptible
Cefazolin	Susceptible
Cefepime	Susceptible
Ceftazidime	Susceptible
Ceftriaxone	Susceptible
Ertapenem	Susceptible
Gentamicin	Susceptible
Imipenem	Susceptible
Meropenem	Susceptible
Piperacillin-tazobactam	Susceptible
Tobramycin	Susceptible
Trimethoprim/sulfamethoxazole	Resistant

a Gatifloxacin breakpoints were considered as the reference for moxifloxacin.

## 3 Discussion

Antibiotic resistance is increasingly compromising the effectiveness of broad spectrum antibiotics, such as fluoroquinolones. Ophthalmic solutions delivered as eye drops result in a peak and trough drug concentration profile, with the trough offering an opportunity for re-establishment of surviving, increasingly resistant microbes. CSLI definition of susceptibility or resistance are usually set for drugs that are administered systemically to treat disseminated disease, and factor in variations in serum levels, organ toxicity, and history of successful infection resolution. The extent to which these definitions translate to conditions achieved during local treatment of the ocular surface remains the subject of considerable controversy.^9^

Although the antibiotic concentration in distinct eye tissue compartments may significantly differ in comparison to systemic levels, the incorporation of pharmacokinetic (PK) and pharmacodynamic (PD) indices to predict treatment efficiency has been recently proposed to overcome the issues of using systemic breakpoints, and support dosing optimization for ocular infections.^9^

Fluoroquinolone antibiotics exert their bactericidal activity in a concentration-dependent manner. The primary PK/PD parameters that are associated with clinical and bacteriological efficacy of these antibiotics are the maximum concentration of antibiotics in the tissue to the MIC ratio (C_max_:MIC) and the 24-h area under the concentration curve to MIC ratio (AUC_0-24_:MIC).^3^ Bactericidal activity of fluoroquinolones increases, as does the antibiotic concentration, so the primary goal of concentration-dependent killing is to maximize the levels of antibiotic in the infected tissue. If these PK/PD goals are not achieved, the clinical and bacteriological efficacy of treatment is compromised and selection of resistant subpopulation is favored.^3^

In the reported case, a PROSE lens was in use for treatment of ocular surface symptoms related to cGVHD. The choice of antibiotic prior to the return of culture results and drug susceptibilities was empiric. The patient was initially treated with topical 0.5% moxifloxacin with no improvement. Although high levels of moxifloxacin are in contact with the ocular surface, a very small fraction of that will penetrate the cornea tissue.^9^ Remarkably, increasing the frequency of the application of the drops via the PROSE reservoir resulted in clearing of the *E. coli* infection, despite the isolated carried multiple QRDR mutations resulting in elevated fluoroquinolone MICs. It seems likely that the PROSE reservoir enhanced the contact time of the drug at the ocular surface and facilitated drug retention, which associated with frequent dosing may significantly increase the antibiotic levels in the cornea tissue. Therefore, PROSE may be taken as an important clinical tool to attain effective PK/PD-based targets for treatment of bacterial keratitis.

*E. coli* is rare as a corneal pathogen. In this case, the infection was likely opportunistic in a cornea with ocular surface compromised by ocular cGVHD, systemic steroid use, use of a PROSE device, corneal limbal stem cell deficiency, dry eye, and diabetes mellitus. This case highlights the potential of the PROSE device to serve as a vehicle for delivering topically applied ocular medications to the cornea. The fluid-ventilated gas-permeable prosthetic device immerses the entire ocular surface in a reservoir of fluid. Oxygenation of the fluid-filled reservoir is principally maintained by oxygen transmission through a gas-permeable polymer material as well as some tear exchange. A PROSE device provides constant lubrication, and the necessary oxygen supply to support healing, reduce symptoms, and improves visual function.^10^

In this complex case of corneal ulcer caused by a fluoroquinolone-resistant *E. coli*, despite the underlying severe cGVHD, topical moxifloxacin was used in conjunction with PROSE treatment, to successfully treat the infection and heal the corneal ulcer. We conclude that frequent dosing to the PROSE reservoir is likely to increase fluoroquinolone levels in the cornea and may represent a valuable tool to overcome antibiotic resistance.

## Patient consent

Written consent to publish this case report has been obtained from the patient.

## Conflicts of interest

None.

## Authorship

All authors attest they meet the current ICMJE criteria for authorship.

## Appendix A Supplementary data

Data Profile Data Profile
